# The Correlation among Neural Dynamic Processing of Conflict Control, Testosterone and Cortisol Levels in 10-Year-Old Children

**DOI:** 10.3389/fpsyg.2017.01037

**Published:** 2017-06-22

**Authors:** Fangfang Shangguan, Tongran Liu, Xiuying Liu, Jiannong Shi

**Affiliations:** ^1^Key Laboratory of Learning and Cognition, Department of Psychology, Capital Normal UniversityBeijing, China; ^2^CAS Key Laboratory of Behavioral Science, Institute of Psychology, Chinese Academy of SciencesBeijing, China; ^3^Department of Psychology, University of Chinese Academy of SciencesBeijing, China

**Keywords:** conflict control, cortisol, testosterone, event-related potentials, children

## Abstract

Cognitive control is related to goal-directed self-regulation abilities, which is fundamental for human development. Conflict control includes the neural processes of conflict monitoring and conflict resolution. Testosterone and cortisol are essential hormones for the development of cognitive functions. However, there are no studies that have investigated the correlation of these two hormones with conflict control in preadolescents. In this study, we aimed to explore whether testosterone, cortisol, and testosterone/cortisol ratio worked differently for preadolescent’s conflict control processes in varied conflict control tasks. Thirty-two 10-year-old children (16 boys and 16 girls) were enrolled. They were instructed to accomplish three conflict control tasks with different conflict dimensions, including the Flanker, Simon, and Stroop tasks, and electrophysiological signals were recorded. Salivary samples were collected from each child. The testosterone and cortisol levels were determined by enzyme-linked immunosorbent assay. The electrophysiological results showed that the incongruent trials induced greater N2/N450 and P3/SP responses than the congruent trials during neural processes of conflict monitoring and conflict resolution in the Flanker and Stroop tasks. The hormonal findings showed that (1) the testosterone/cortisol ratio was correlated with conflict control accuracy and conflict resolution in the Flanker task; (2) the testosterone level was associated with conflict control performance and neural processing of conflict resolution in the Stroop task; (3) the cortisol level was correlated with conflict control performance and neural processing of conflict monitoring in the Simon task. In conclusion, in 10-year-old children, the fewer processes a task needs, the more likely there is an association between the T/C ratios and the behavioral and brain response, and the dual-hormone effects on conflict resolution may be testosterone-driven in the Stroop and Flanker tasks.

## Introduction

Conflict control is essential for goal-directed behavior and self-regulation (Mayr et al., 2003; Botvinick et al., 2004; Bexkens et al., 2014). It requires individuals to concentrate on the instructed mental operation and filter out distraction (Nigg, 2000; Johnstone et al., 2009; Mullane et al., 2009). Conflict control is known to develop rapidly during early childhood and continues to develop during adolescence (Rueda et al., 2004; Davidson et al., 2006; Tsujimoto, 2008; van Meel et al., 2012; Sheridan et al., 2014). According to the dimensional overlap theory (Kornblum et al., 1990, 1999), conflict control processes are investigated using the Flanker task (Eriksen and Eriksen, 1974), Stroop task (Stroop, 1935), and Simon task (Simon and Small, 1969). The Flanker task examines the stimulus–stimulus (S–S) incompatibility. The Simon task requires attentional control on the conflicts stemming from stimulus–response (S–R) incompatibility (Proctor and Reeve, 1990). The Stroop task measures the mixed S–S and S–R incompatibilities (Liu et al., 2010). S–S and S–R conflict control have been shown to develop differently with different developmental speeds and patterns (Diamond and Taylor, 1996; Jongen and Jonkman, 2008; Bryce et al., 2011). In addition, executive functions have been reported to be indistinguishable in children until 9 years of age and are related yet separable by 10- to 11-years-old (Brydges et al., 2014b).

Conflict control contains two sub-processes: conflict monitoring and conflict resolution (van der Molen, 2000; Botvinick et al., 2004; Kerns et al., 2004; Rueda et al., 2004; Polich, 2007; Hillman et al., 2009; Johnstone et al., 2009; Frühholz et al., 2011; Brydges et al., 2014a; Wang et al., 2014). Conflict monitoring is the detection of conflicts coming from perceptual inputs or between preferred responses and required responses. The frontal N2 and N450 components of event-related potentials (ERPs) reflect the neural processing of conflict monitoring (van Veen and Carter, 2002; Botvinick et al., 2004; Kerns et al., 2004; Tillman and Wiens, 2011; Brydges et al., 2013; Larson et al., 2014). Conflict resolution is associated with the neural processing of resolution of the conflicts. The positive P3 and slow potential (SP) with central-parietal distribution correlate with conflict resolution (Liotti et al., 2000; Rueda et al., 2004; Jonkman, 2006; Smith et al., 2008; Abundis-Gutierrez et al., 2014).

Preadolescence is a critical period for the development of conflict control (Khan et al., 2015; Crone and Steinbeis, 2017), and the immature prefrontal cognitive control is related with a range of adverse outcomes in preadolescence and adolescence periods, such as smoking, alcohol/drug abuse, risking behaviors (see Pfeifer and Allen, 2012 for a review). Steroid hormones have been shown to affect the cerebral cortex in the preadolescent period (Eiland and Romeo, 2013; Nguyen et al., 2013). Testosterone is the most commonly studied hormone. The first significant rise in testosterone occurs at 10 years in both chronological and bone age (SizoNenko and Paunier, 1975). Studies have shown that testosterone reduces fear (Hermans et al., 2006) and predicts risk-seeking behavior (Sapienza et al., 2009; Stanton et al., 2011). Moreover, testosterone is considered to be an important factor during brain development of cognition (Janowsky, 2006), and testosterone level is associated with several cognitive functions (for reviews, Moffat, 2005; Janowsky, 2006; Driscoll and Resnick, 2007), especially executive function (Barrett-Connor et al., 1999; Perry et al., 2001; Muller et al., 2005). The testosterone level moderates cognitive function by attentional control processes in older men (Martin et al., 2009). The level of testosterone is inconsistently correlated with conflict control in the Flanker and Stroop tasks. Participants with higher testosterone levels have been reported to exhibit more interference than those with lower testosterone levels in the Flanker task (van Strien et al., 2009). In contrast, testosterone deprivation showed an adverse effect on Stroop interference performance (Green et al., 2004).

Another important hormone is cortisol, and high cortisol level is associated with psychological stress and behavioral inhibition in both child and adult participants (Fox et al., 2005; Terburg et al., 2009; Tops and Boksem, 2011; Ventura et al., 2012; Pfattheicher and Keller, 2014). There are two types of cortisol effects. One is the slow, genomic effect, which is brought about by modulation of gene expression; and the other is the rapid-acting, non-genomic cortisol effect without modulation of gene expression (Joëls et al., 2011). Shields et al. (2015) proposed, on the basis of meta-analysis, that the rapid, non-genomic effects of cortisol enhanced inhibition, but the slow, genomic effects of cortisol impaired inhibition. They suggested that cortisol might act in concert with other biological processes to further influence core executive functions.

Terburg et al. (2009) proposed a dual-hormone hypothesis that testosterone might interact with cortisol to affect behavioral regulation (Archer, 2006; Mehta and Josephs, 2010; Mehta et al., 2015; Ponzi et al., 2016; Sherman et al., 2016). According to the testosterone/cortisol (T/C) ratio hypothesis, high testosterone in the presence of low cortisol is associated with aggression and externalizing problems in adults through up-regulation of gene expression in several brain regions including the amygdala (van Honk et al., 2010; Montoya et al., 2012). Increased testosterone relative to cortisol may reduce the communication between subcortical regions (such as the amygdala) and cortical regions (such as orbitofrontal cortex) (Glenn et al., 2011), and the decoupling between subcortical and cortical regions may have effects on emotional information processing (van Honk and Schutter, 2006). However, there is little evidence about the association between non-emotional information processing and T/C ratio, except for a few studies concerning testosterone or cortisol and cognitive functions such as processing speed, attention (Salminen et al., 2004; Henry and Sherwin, 2012), and cognitive control mentioned above.

Although testosterone and cortisol have both been reported to be correlated with cognitive control processes, it is unknown how the T/C ratio impacts cognitive control. During the transition period from childhood to adolescence, testosterone and cortisol secretion experience a transition from adrenarche to gonadarche. In addition, it has been shown that morning salivary cortisol levels in mid-postpubertal girls are greater than in mid-postpubertal boys, but it is not the case in pre-early pubertal girls and boys (Netherton et al., 2004). Little attention has been paid to whether the surge of hormone secretion correlates with conflict control during the transition, despite that the target brain regions of these hormones overlap with the neural basis of conflict control.

In this study, we explored the correlation of testosterone and cortisol levels with conflict control processes in preadolescent children. We hypothesized that testosterone, cortisol, and the T/C ratio are differently correlated with children’s conflict control performance and neural processing of conflict monitoring and conflict resolution. We also expected that testosterone and cortisol would work differently for S–S, S–R, and mixed S–R types of conflicts, due to the varied electrophysiological activity in the Flanker, Simon, and Stroop tasks. This is the first study that demonstrates the dual-hormone regulation of conflict control in preadolescent children.

## Materials and Methods

### Subjects

Thirty-two children (16 boys and 16 girls, ages from 10.1 to 11.2 years, mean age: 10.8 ± 0.4 years) were randomly enrolled from one elementary school. The enrollment of participants was in agreement with the Declaration of Helsinki. This study was approved by the Ethics Committee of Chinese Academy of Sciences and Capital Normal University. Written informed consents were obtained from all of the children and their parents. All participants were right-handed with normal visual acuity and without any neurological or psychiatric problems. They were all naïve to the purposes of the experiment.

### Materials and Procedures

**FIGURE 1:**
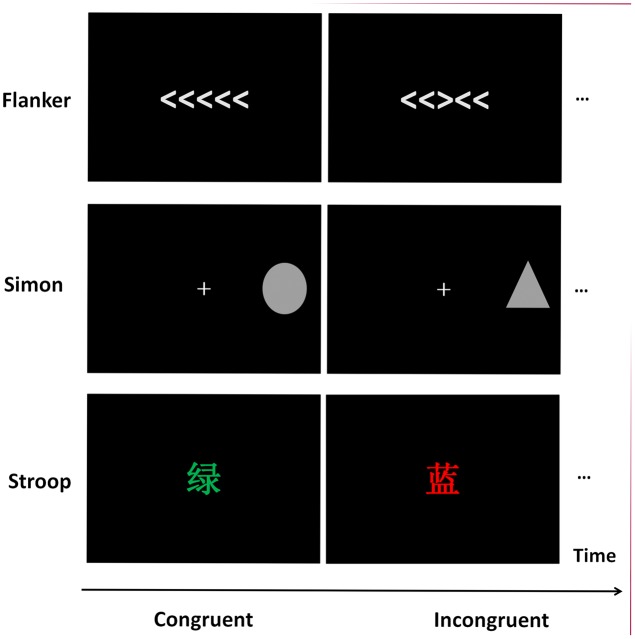
The samples of stimuli in Flanker, Simon, and Stroop tasks.

The stimuli and procedures used are illustrated in **Figure 1**. The presentation screen was a computer monitor (17 inches, 1024 × 768 at 100 Hz) with a black background, and the participant’s viewing distance was 60 cm. The participants were instructed that they would perform a Simon task, a Flanker task, and a Stroop task, and the sequences of the presentation of these three tasks were counterbalanced among participants.

#### Flanker Task

A revised Flanker task was adopted, and each stimulus contained five arrows in a row (visual angle was approximately 8.0° × 1.2°). Each trial began with a fixation cross for 250 ms, followed by the presentation of the stimulus for 1500 ms. The inter-stimulus interval (ISI) varied randomly between 800 and 1000 ms. In congruent trials, the pointing direction of the central target arrow was the same as the bilateral four arrows, whereas in incongruent trials, the central target arrow pointed opposite to the direction of the other arrows. Participants were instructed to press the right button with the right index finger if the target arrow pointed to the right and press the left button with the left index finger if the target arrow pointed to the left.

Before the main experiment, the participants were given 16 practice trials to familiarize themselves with the task procedure. The formal experiment contained 4 blocks with 61 trials in each block, and the total 244 trials consisted of 122 congruent trials and 122 incongruent trials. A 2- to 3-min rest period was given after each block, and the whole task lasted approximately 15 min.

#### Simon Task

A revised Simon task (Simon, 1969; Hommel, 1993) was adopted. For each trial, either a gray triangle or a gray square was displayed on the left half side or the right half side of the black screen, with a visual angle of 1.2° vertically and 1.2° horizontally. Each trial began with a fixation cross for 250 ms, followed by the presentation of the stimulus for 1500 ms. The ISI varied randomly between 800 and 1000 ms. Participants were instructed to press the left button with the left index finger and the right button with the right index finger for the triangle stimulus or the square stimulus. The response hands for the corresponding stimuli were balanced among participants. For the congruent trials, the mapping between the stimulus presentation location and the response hand was compatible; while, for the incongruent trials, the stimulus–response mapping was incompatible.

Before the main experiment, the participants were given 16 practice trials to familiarize themselves with the task procedure. The formal experiment contained 4 blocks with 61 trials in each block, and the total 244 trials consisted of 122 congruent trials and 122 incongruent trials. A 2- to 3-min rest period was given after each block, and the whole task lasted approximately 15 min.

#### Stroop Task

A revised Stroop task was adopted (Spillers and Unsworth, 2011; Unsworth et al., 2011), and Chinese characters, “
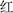
”(red), “
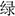
”(green), and “
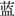
”(blue), with either red, green or blue ink were displayed at the center of the black screen. The visual angle of the stimuli was 1.2° vertically and 1.2° horizontally. Each trial began with a fixation cross for 250 ms, followed by the presentation of the stimulus for 1500 ms. The ISI varied randomly between 800 and 1000 ms. Participants were required to press three buttons according to the ink color of the stimuli with their right index, middle, and ring finger, and ignore the meaning of the characters. For the congruent trials, the ink color of the stimuli was compatible with the meanings of the words, while for the incongruent trials, the ink color of the stimuli was incompatible with the meanings of the words. The response fingers for the corresponding stimuli were balanced among participants.

Before the main experiment, the participants were given 24 practice trials to familiarize themselves with the task procedure. The formal experiment contained 4 blocks with 81 trials in each block, which consisted of 162 congruent trials and 162 incongruent trials in total. A 2- to 3-min rest period was given after each block, and the whole task lasted approximately 20 min.

### Saliva Sample Collection and Measurement

The salivary samples were collected a week after the ERP experiments, and the salivary samples of all the children were collected at the same time of the same day. Each child was asked to rinse his/her mouth thoroughly three times with water at 8 am. Then, 2 mL saliva was collected from each child at approximately 9 am. Subjects were asked not to eat or drink during the interval. Saliva was collected by using the Salivette device. Each child was instructed to keep the sponge in his/her mouth and chew and roll the sponge gently inside the mouth for 2 min. Samples were stored at -20°C. The levels of salivary testosterone and cortisol were measured by enzyme-linked immunosorbent assay (ELISA). The Kits used for ELISA were from DRG Instruments GmbH, Germany.

### EEG Recording and Analysis

Sixty-four electrodes embedded in a NeuroScan Quik-Cap were used to record the electroencephalograms (EEG), and the electrode positions were placed according to the 10–20 system locations. Four bipolar electrodes were positioned on the inferior and superior areas of the left eye and the outer canthi of both eyes to monitor the vertical and horizontal EOG (VEOG and HEOG). Electrode impedance was kept below 5 kΩ. The EEG signal was amplified using SynAmps amplifiers with a sample rate of 1000 Hz and was continuously recorded with online bandpass filters at 0.05–100 Hz with a nose reference. The epochs contaminated by eye blinks, eye movements, or muscle potentials exceeding ±70 μV at any electrode were excluded using the SCAN program. The laboratory had efficient decoration to shield ambient electrical noise. The signal was further epoched with 100 ms prior to (for baseline correction) and 1000 ms after the stimulus onset. ERPs were further zero phase shift digitally filtered off-line (bandwidth: 1–30 Hz, slope: 24 dB/octave).

Based on previous neurodevelopment literature on conflict control (Rueda et al., 2004) and current data, the N2 (with a 220–340-ms time window; the electrodes of F3, FC3, and C3 were averaged for the left hemisphere; the average of Fz, FCz, and Cz for the midline area; the average of F4, FC4, and C4 for the right hemisphere) and P3 components (with a 350–530-ms time window; the electrodes of C3, CP3, and P3 were averaged for the left hemisphere; the average of Cz, CPz, and Pz for the midline area; the average of C4, CP4, and P4 for the right hemisphere) were analyzed for the Flanker and Simon tasks. The N450 (400–500-ms time window; the electrodes of F3, FC3, and C3 were averaged for the left hemisphere; the average of Fz, FCz, and Cz for the midline area; the average of F4, FC4, and C4 for the right hemisphere) and SP (570–770-ms time window; the electrodes of C3, CP3, and P3 were averaged for the left hemisphere; the average of Cz, CPz, and Pz for the midline area; the average of C4, CP4, and P4 for the right hemisphere) components were analyzed for the Stroop task.

### Statistical Analysis

For the behavioral performances, the dependent factors of mean accuracy and median of reaction time (RT) were tested singly with the 2 × 2 repeated ANOVAs in the Flanker task, Simon task, and Stroop task, separately, and each ANOVA had two independent factors of Congruency (congruent and incongruent) and Sex (boy and girl). For electrophysiological activities, the dependent factors of N2 peak latencies, N2 mean amplitudes, P3 peak latencies, and P3 mean amplitudes were tested singly with the 2 × 3 × 2 × 2 repeated ANOVAs, and all the ANOVAs had four independent factors of Congruency (congruent, incongruent), Hemisphere (left, middle, right), Task (Flanker, Simon), and Sex (boy, girl). The dependent factors of N450 mean amplitudes and SP mean amplitudes were tested singly with the 2 × 3 × 2 repeated ANOVAs, and both of the ANOVAs had three independent factors of Congruency (congruent and incongruent), Hemisphere (left, middle, and right), and Sex (boy and girl). Greenhouse-Geisser correction for violations of sphericity was used where appropriate. The *post hoc* contrasts with Bonferroni correction for multiple comparisons were conducted for significant main and interaction effects, and the *post hoc* comparisons would be carried out to compare between all levels.

Correlation analyses were further conducted among hormone levels (testosterone levels, cortisol levels, and T/C ratio), behavioral responses (median RT and accuracy), and electrophysiological processes (latency and amplitudes of ERP responses) of conflict control in the three tasks. Pearson analysis was conducted when performing a correlation analysis between two normally distributed variables. Spearman analysis was conducted when the distributions of the two variables were not both normally distributed.

## Results

### Salivary Testosterone and Cortisol Levels

To explore the hormone regulation of conflict control, we first measured the testosterone and cortisol levels in 10-year-old children. The salivary testosterone levels were 18.86 ± 6.56 in boys and 25.97 ± 14.82 in girls (*p* > 0.05); the salivary cortisol levels were 2.59 ± 0.67 in boys and 3.03 ± 0.87 in girls (*p* > 0.05); and the testosterone/cortisol ratios were 7.40 ± 2.20 in boys and 8.60 ± 3.03 in girls (*p* > 0.05).

### Behavior Performance on the Three Tasks

The mean accuracies and median RTs in congruent and incongruent trials of all the three tasks are shown in **Table 1**.

**Table 1:** Mean accuracy and median of reaction time (ms) in all the tasks.

	Congruent		Incongruent	

	**Reaction time**	**Accuracy**	**Reaction time**	**Accuracy**
Flanker	460 (57)	0.98 (0.02)	489 (66)	0.97 (0.02)
Simon	541 (77)	0.94 (0.04)	547 (76)	0.93 (0.04)
Stroop	642 (63)	0.95 (0.05)	724 (93)	0.89 (0.07)

#### Median RT and Accuracy of Responses in the Flanker Task

For the RT in the Flanker task, the main effect of Congruency was significant [*F*(1,30) = 49.0, *p* < 0.001, η^2^ = 0.62], and participants had shorter RT in congruent trials than in incongruent trials.

With regard to the accuracy in the Flanker task, the Congruency factor showed a significant main effect [*F*(1,30) = 9.9, *p* < 0.005, η^2^ = 0.24], and children showed higher accuracy in congruent trails than in incongruent trials.

#### Median RT and Accuracy of Responses in the Simon Task

No significant main or interaction effects were found for median RT or accuracy in the Simon task (*p*s > 0.05).

#### Median RT and Accuracy of Responses in the Stroop Task

For the RT in the Stroop task, the main effect of Congruency was significant [*F*(1,30) = 93.5, *p* < 0.001, η^2^ = 0.76], and participants had shorter RT in congruent trials than in incongruent trials.

With regard to the accuracy in the Stroop task, the Congruency factor showed a significant main effect [*F*(1,30) = 6.3, *p* < 0.05, η^2^ = 0.17], and children showed higher accuracy in congruent trails than in incongruent trials.

### ERP Waveforms

**FIGURE 2:**
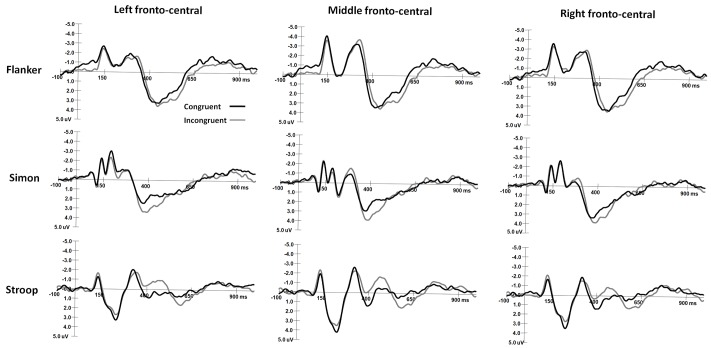
The grand-average waveforms of N2 components in Flanker and Simon tasks and the N450 component in Stroop task.

**FIGURE 3:**
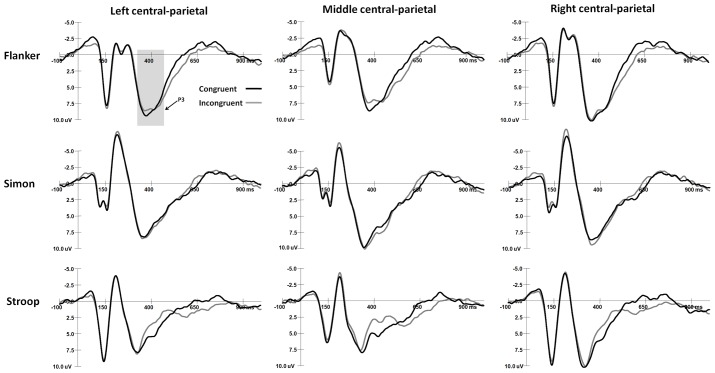
The grand-average waveforms of P3 components in Flanker and Simon tasks and the SP component in Stroop task.

The mean peak latency and amplitude of the N2 and P3 components in the Simon and Flanker tasks are shown in **Table 2**. The grand-average ERP waveforms of the N2 and N450 components are shown in **Figure 2**, and the grand-average ERP waveforms of the P3 and SP components are shown in **Figure 3**. The waveforms are described in detail as follows.

**Table 2:** Mean latencies (ms) and amplitudes (μV) of N2 and P3 components in all the conditions.

	Congruent				Incongruent			
	N2		P3		N2		P3	
	Latency	Amplitude	Latency	Amplitude	Latency	Amplitude	Latency	Amplitude
Flanker	275	-5.05	414 (32)	10.03 (2.83)	278	-5.52	429 (33)	9.95 (2.57)
Simon	266	-3.87	382 (26)	10.19 (3.47)	269	-3.91	382 (25)	10.54 (3.60)

#### N2 Responses in the Flanker and Simon Tasks

For the ANOVA of N2 latencies, the main effect of Task was significant [*F*(1,30) = 4.55, *p* < 0.05, η^2^ = 0.13], and N2 latencies were shorter in the Simon task than in the Flanker task. The main effect of Hemisphere was significant [*F*(2,60) = 8.91, *p* < 0.001, η^2^ = 0.23], and N2 latencies were longer over midline area than over the left and right hemispheres (*p*s < 0.005). The main effect of Sex on N2 latencies was also significant [*F*(1,30) = 4.67, *p* < 0.05, η^2^ = 0.14], and girls had shorter N2 latencies than boys did. No other significant main or interaction effects were observed for N2 latencies (*p*s > 0.05).

For the ANOVA of N2 amplitudes, the interaction between Hemisphere and Sex was significant [*F*(2,60) = 7.31, *p* < 0.001, η^2^ = 0.20], and girls had more negative N2 amplitudes over the right hemisphere than over the left hemisphere (*p* < 0.05) and midline area (*p* < 0.005); no other comparisons were found to be significant (*p*s > 0.05). The interaction between Task, Congruency, and Hemisphere was significant [*F*(2,60) = 3.52, *p* < 0.05, η^2^ = 0.11], and N2 responses were more negative in the Flanker task than in the Simon task over the midline and right hemisphere on congruent trials (midline: *p* < 0.01; right: *p* < 0.05) and incongruent trials (midline: *p* < 0.001; right: *p* < 0.01). N2 was more negative on incongruent trials than on congruent trials in the Flanker task over the midline area (*p* < 0.05). In the Flanker task, N2 amplitudes were more negative over the midline area than over the left and right hemispheres on both congruent and incongruent trials (*p*s < 0.005); in the Simon task, N2 amplitudes were more negative over the left hemisphere than over the midline area on congruent trials (*p* < 0.01). No other significant main effects or interaction effects were observed for N2 amplitudes (*p*s > 0.05).

#### N450 Responses in the Stroop Tasks

N450 amplitudes were 1.36 ± 5.08 μV on congruent trials and -0.01 ± 4.49 μV on incongruent trials. For the ANOVA of the N450 amplitudes, the main effect of Congruency was significant [*F*(1,30) = 13.57, *p* < 0.001, η^2^ = 0.33], and the N450 was more negative on incongruent trials than on congruent trials. The interaction between Sex and Hemisphere was significant [*F*(2,60) = 3.30, *p* < 0.05, η^2^ = 0.11], and the N450 of girls was more negative over the midline area than over the left hemisphere (*p* < 0.05); no other comparisons were found to be significant (*p*s > 0.05). No other significant main effects or interaction effects were observed for N450 amplitudes (*p*s > 0.05).

#### P3 Responses in the Flanker and Simon Tasks

For the ANOVA of P3 latencies, the main effect of Task was significant [*F*(1,30) = 55.56, *p* < 0.001, η^2^ = 0.65], and the P3 latency was shorter in the Simon task than in the Flanker task. The main effect of Sex was significant [*F*(1,30) = 4.62, *p* < 0.05, η^2^ = 0.13], and girls had shorter P3 responses than boys did. The interaction between Task and Congruency was significant [*F*(1,30) = 9.24, *p* < 0.005, η^2^ = 0.24], and in the Flanker task, the P3 responses were faster on congruent trials than on incongruent trials (*p* < 0.001); no other comparisons were found to be significant (*p*s > 0.05). No other significant main effects or interaction effects were observed for P3 latencies (*p*s > 0.05).

For the ANOVA of P3 amplitudes, the interaction between Congruency and Sex was significant [*F*(1,30) = 4.93, *p* < 0.05, η^2^ = 0.14], and girls exhibited a stronger P3 on congruent trials than on incongruent trials (*p* < 0.05); no other comparisons were found to be significant (*p*s > 0.05).The interaction between Task and Hemisphere was significant [*F*(2,60) = 12.00, *p* < 0.001, η^2^ = 0.29], and the P3 was stronger in the Simon task than in the Flanker task over the midline area (*p* < 0.05); moreover, in the Simon task, the P3 was stronger over the midline area and the right hemisphere than over the left hemisphere (midline: *p* < 0.001; right: *p* < 0.05). No other significant main effects or interaction effects were observed for P3 amplitudes (*p*s > 0.05).

#### SP Responses in the Stroop Tasks

The SP amplitudes were 0.23 ± 1.33 μV on congruent trials and 1.49 ± 1.56 μV on incongruent trials. For the ANOVA of SP amplitude, the main effect of Congruency was significant [*F*(1,30) = 18.36, *p* < 0.001, η^2^ = 0.40], and the SP was stronger in incongruent trials than in congruent trials. The interaction between Congruency and Hemisphere was significant [*F*(2,60) = 4.10, *p* < 0.05, η^2^ = 0.13], and in congruent trials, the SP was stronger over the midline area than over the right hemisphere (*p* < 0.01); no other comparisons were found to be significant (*p*s > 0.05). The interaction among Sex, Congruency, and Hemisphere was significant [*F*(2,60) = 3.84, *p* < 0.05, η^2^ = 0.12], and in congruent trials, boys had a stronger SP than girls did over the left hemisphere and midline area (left: *p* < 0.01, midline: *p* < 0.05); no other comparisons were found to be significant (*p*s > 0.05). No other significant main effects or interaction effects were observed for SP amplitudes (*p*s > 0.05).

### The Correlation among Behavior, ERP, and Hormone Levels

The results of the correlation analyses among the behavioral performance (median RT and accuracy), ERP (latency and amplitude of ERP components), testosterone and cortisol levels, and the T/C ratios are displayed in **Table 3**. Cortisol levels were negatively correlated with RTs in the Simon task for both congruent (*r* = -0.45, *p* < 0.05) and incongruent trials (*r* = -0.42, *p* < 0.05). Testosterone (*r* = -0.53, *p* < 0.01) and cortisol (*r* = -0.41, *p* < 0.05) levels were both negatively correlated with the dRTs (RT differences between incongruent and congruent trials) in the Stroop task. Testosterone and cortisol levels were also negatively correlated with the RTs in the Stroop task for both congruent (testosterone: *r* = -0.38, *p* < 0.05; cortisol: *r* = -0.48, *p* < 0.01) and incongruent trials (testosterone: *r* = -0.54, *p* < 0.01; cortisol: *r* = -0.54, *p* < 0.05). For accuracy, the T/C ratios were negatively correlated with accuracy in the Flanker task for both congruent (*r* = -0.40, *p* < 0.05) and incongruent (*r* = -0.54, *p* < 0.05) trials. Testosterone levels were also negatively correlated with accuracy in the Flanker task for congruent trials (*r* = -0.39, *p* < 0.05).

**Table 3:** The correlations among behavioral data, ERP data, and hormone levels.

			Testosterone	Cortisol	T/C effect
**Median of reaction time**	Flanker	C	-0.15	-0.34	0.10
		I	-0.14	-0.35	0.12
		I–C	-0.04	-0.18	0.10
	Simon	C	-0.27	-0.45*	0.05
		I	-0.29	-0.42*	0.002
		I–C	-0.02	0.08	-0.10
	Stroop	C	-0.38*	-0.48**	-0.07
		I	-0.54**	-0.54**	-0.21
		I–C	-0.53**	-0.41*	-0.31
**Mean accuracy**	Flanker	C	-0.39*	0.14	-0.54**
		I	-0.34	-0.01	-0.40*
		I–C	0.02	-0.15	0.07
	Simon	C	-0.17	0.15	-0.36*
		I	-0.10	0.05	-0.08
		I–C	0.07	-0.07	0.18
	Stroop	C	-0.11	0.02	-0.10
		I	-0.02	0.14	0.06
		I–C	0.14	0.08	0.22
**N2 latency**	Flanker	C	0.01	0.10	0.02
		I	0.03	0.08	-0.11
		I–C	-0.15	-0.01	-0.18
	Simon	C	0.03	-0.07	0.03
		I	-0.03	-0.16	0.11
		I–C	0.01	-0.36*	0.32
**N2 amplitude**	Flanker	C	0.27	0.13	0.24
		I	0.26	0.22	0.09
		I–C	-0.17	0.09	-0.26
	Simon	C	0.02	0.16	-0.11
		I	0.06	0.15	-0.04
		I–C	0.16	0.00	0.11
**N450 amplitude**	Stroop	C	-0.01	0.06	-0.17
		I	0.05	0.07	0.01
		I–C	-0.11	0.01	0.18
**P3 latency**	Flanker	C	-0.15	0.17	-0.30
		I	-0.12	0.13	-0.40*
		I–C	-0.09	-0.06	-0.19
	Simon	C	0.06	0.15	-0.13
		I	0.07	0.10	-0.11
		I–C	-0.07	-0.13	-0.02
**P3 amplitude**	Flanker	C	-0.09	-0.25	0.00
		I	-0.03	-0.22	0.02
		I–C	0.11	0.11	0.02
	Simon	C	-0.07	-0.04	-0.02
		I	-0.10	-0.03	0.04
		I–C	-0.02	0.01	0.10
**SP amplitude**	Stroop	C	-0.43*	-0.29	-0.14
		I	-0.20	-0.05	-0.11
		I–C	0.23	0.21	-0.02

*RT, reaction time, C, congruent, I, incongruent, I–C = incongruent–congruent. ^∗^*p* < 0.05; ^∗∗^*p* < 0.01*.

For the correlation between neural processing of conflict monitoring and the T/C ratios, dN2 latencies (N2 latency differences between incongruent and congruent trials) in the Simon task were negatively correlated with cortisol levels (*r* = -0.36, *p* < 0.05). The N2 amplitude over the midline area on congruent trials of the Flanker task was positively correlated with testosterone levels (*r* = 0.38, *p* < 0.05). Additionally, the dN450 amplitudes (N450 amplitude differences between incongruent and congruent trials) in the Stroop task over the right hemisphere were positively correlated with testosterone levels (*r* = 0.40, *p* < 0.05).

For the correlation between the P3 and SP neural responses for conflict resolution and the T/C ratios, the P3 latencies over the midline area in the Flanker task were negatively correlated with the T/C ratios for both congruent (*r* = -0.41, *p* < 0.05) and incongruent (*r* = -0.47, *p* < 0.01) trials. The SP amplitudes on congruent trials in the Stroop task over the midline area (*r* = -0.37, *p* < 0.05) and left hemisphere (*r* = -0.41, *p* < 0.05) were both negatively correlated with cortisol levels. Testosterone levels were negatively correlated with the SP amplitudes on congruent trials of the Stroop task (*r* = -0.43, *p* < 0.05).

## Discussion

The current study investigated the correlations between hormonal levels and conflict control processes in the flanker, Simon, and Stroop tasks. It was observed that the testosterone levels related with conflict control in Stroop task, the cortisol levels with that in Simon task, and T/C ratio with flanker task, which might support the dimensional overlap theory of conflict control (Kornblum et al., 1990, 1999). Our main findings revealed that for both S–S and S–R conflict control, testosterone and cortisol, alone or jointly, were negatively correlated with the efficiency of conflict control processes indexed by response speed and accuracy. The T/C ratios, testosterone, and cortisol levels were correlated with behavioral efficiency and brain activities during conflict monitoring and resolution process in different ways.

### Dual Hormones and Behavioral Efficiency of Conflict Control

The current study found that, consistent with the results from Green et al. (2004), in the Stroop task, higher testosterone levels were correlated with smaller RT differences between incongruent and congruent trials. The associations between RT differences and cortisol levels were in the same direction as testosterone levels for this instance, and cortisol levels were negatively correlated with the RT differences between incongruent and congruent trials in the Stroop task. It was currently observed that RTs were shorter and accuracies were higher in congruent trials than in incongruent trials in the Flanker and Stroop tasks, and these findings were consistent with previous studies (Perlstein et al., 1998; Botvinick et al., 2004; Rueda et al., 2004; Crump et al., 2006). The three tasks vary in cognitive load of stimulus processing and response selection, as well as in the nature of the conflict, such as S–S, S–R or the mixture of both. The Stroop task requires more processes than the Simon and Flanker tasks (Kornblum, 1994; Liu et al., 2010). Thus, the associations between lower dual-hormone levels and poorer efficiency in the RTs in the Stroop task are consistent with previous evidence that lower testosterone was associated with decreased performance in tasks requiring complex information processing (Green et al., 2004). Additionally, higher cortisol levels were also associated with a slower response speed in the Simon task, which is in line with the observation that cortisol levels are negatively correlated with advanced executive functions (van Honk et al., 2003; Oei et al., 2006; Schoofs et al., 2008; Shields et al., 2015).

In the current study, the behavioral response speed in the Flanker task showed no significant correlations with testosterone. However, in another two previous studies, higher testosterone levels were observed to be associated with a faster response speed in congruent Flanker trials in older men (van Strien et al., 2009), and testosterone deprivation was linked to decreased cognitive processing speed in attentional and working memory tasks in older men (Salminen et al., 2004). The inconsistencies among these findings indicate that the effects of testosterone on brain regions may be different between children and seniors.

### Hormones and Neural Processing of Conflict Monitoring

During neural processing of conflict monitoring, testosterone levels were positively associated with congruency effects in N450 amplitudes over the right hemisphere in the Stroop task, while cortisol levels negatively correlated with congruency effects in the Simon task. These correlations can be explained by the different conflict processings in S–S and S–R conflict control tasks.

In this study, we found that incongruent trials induced more negative N2/N450 responses than congruent trials in the Flanker and Stroop tasks during neural processing of conflict monitoring. The present N2 findings suggest that it took children faster neural speed and less neural effort to detect S–R conflicts (in the Simon task) than to detect S–S conflicts (in the Flanker task), further supporting the dimensional overlap theory for stimulus–stimulus and stimulus–response incompatibility effects (Kornblum et al., 1990, 1999), and demonstrated the independence of stimulus–response incompatibility effects during conflict monitoring processing (Treccani et al., 2009; Liu et al., 2010). Previous evidence suggested that adolescents with higher cortisol levels showed reduced activation compared to that of controls in the right inferior frontal cortex and dorsolateral prefrontal cortex in the comparison of incongruent–congruent trials in the Simon task (Pruessner et al., 2003; Halari et al., 2009). The negative correlation between cortisol levels and frontal cortex activation in the Simon task may integrate the whole conflict processing during S–R conflict control, but the N2 latency in the current study might mainly reflect faster conflict detection on S–R conflicts, which led to a different cortisol effect in the Simon task. In the Stroop task, stimulus processing partly relies on semantic features; so the conflict monitoring may be more tightly related with testosterone because of semantic processing (Geschwind and Galaburda, 1985). The positive correlation between testosterone and congruency effects over the right hemisphere in the Stroop task during conflict monitoring reflected that testosterone enhanced the cognitive effort in the right hemisphere to detect conflicts coming from perceptual inputs (ink color and meanings of the characters, S–S) or between preferred responses and required responses (S–R). For the less complex task, the Flanker task, no correlations between hormone levels and congruency effects were found, but testosterone levels were positively correlated with the N2 amplitudes in congruent trials over the midline area.

### Hormones and Neural Processing of Conflict Resolution

In this study, we also found that P3/SP responses were stronger and slower on incongruent trials than in congruent trials of the Stroop and Flanker tasks during neural processing of conflict resolution, which is consistent with cognitive control theory and prior empirical findings (Liotti et al., 2000; Rueda et al., 2004; Jonkman, 2006; Smith et al., 2008; Abundis-Gutierrez et al., 2014). Hormone effects on P3/SP responses reflected a possible testosterone-driven pattern in the Stroop and Flanker tasks.

In the Stroop task, cortisol levels were negatively correlated with SP amplitude over the midline area and the left hemisphere on the congruent trials, while testosterone levels were positively associated with the SP amplitudes overall on the congruent trials. These results are consistent with previous evidence that cortisol might exert negative effects on response selection, namely, response inhibition (Fox et al., 2005; Tops et al., 2006; Shields et al., 2015), while testosterone reduces impulse control (Bing et al., 1998) and might be related to stronger cortex activation. According to the current results, cortisol reduced the neural effort of conflict resolution over the midline area and the left hemisphere of central and parietal areas, while testosterone enhanced the neural effort of conflict resolution over the whole central and parietal cortex. Furthermore, higher levels of the two hormones were related to higher efficiency in the RTs in the Stroop task. In addition, testosterone enhanced both conflict monitoring and resolution, whereas in contrast, cortisol was only related to conflict resolution. Thus, despite the relationship to cortisol, the congruency effects observed in the RTs in the Stroop task seemed to be mostly driven by testosterone, which is in accordance with previous evidence of testosterone-driven HPA-HPG coupling (Dismukes et al., 2015).

An important finding in the Flanker task was that the T/C ratio was negatively related to the neural processing speed of conflict resolution over the midline area in both incongruent and congruent trials. A previous study reported that in the Flanker task, cortisol mobilization was positively correlated with the error-related negativity (ERN) responses, and a non-significant correlation was observed between cortisol and the N2 and P3 responses for conflict monitoring and conflict resolution processes (Tops et al., 2006). Our current findings supported these findings that cortisol levels alone were not correlated with both the N2 and P3 responses in the Flanker task, which further revealed that testosterone and cortisol worked together to affect conflict control performance and neural processing of conflict resolution. The T/C ratios and testosterone levels were negatively related with accuracy in the Flanker task; this might have mainly been due to the trade-off between the conflict resolution speed and behavioral response accuracy, because testosterone seemed to play a positive role in conflict monitoring of S–S conflicts. Thus, the dual-hormone effects on performance in the Flanker task as indexed by accuracy may also be mostly driven by testosterone (Dismukes et al., 2015).

It seems that the fewer processes a task requires, the more likely there are associations between the T/C ratios and responses on the task. Secondly, the T/C ratios may not be correlated with executive functions that are relatively underdeveloped. According to the dimensional overlap theory, the Flanker task required fewer processes than the Simon and Stroop tasks, and in every task, congruent trials required fewer processes than incongruent trials. In the current study, the T/C ratios were correlated with N2 and P3 in the Flanker task but not in the other two tasks. What’s more, response interference control has been reported to be a later developed ability (Jongen and Jonkman, 2008; Bryce et al., 2011). The behavioral response speed in the Stroop and Simon tasks was significantly correlated with testosterone or cortisol levels separately, but not with the T/C ratios. Since testosterone has been reported to correlate with cognitive development in pre-early adolescents (Shangguan and Shi, 2009), the T/C ratio should be considered as a critical factor in the development of cognitive control, especially from late childhood to adolescence.

## Conclusion

This study advanced our knowledge of hormone-brain-behavior associations in cognitive conflict control. We found that testosterone, cortisol, and the T/C ratio were separately related to conflict control processes for varied conflicts in 10-year-old children. Cortisol levels were associated with conflict control performance and neural processing of conflict monitoring on stimulus–response conflicts in the Simon task. Testosterone levels were tightly correlated with conflict control performance and neural processing of conflict monitoring in the Stroop task. The T/C ratios were correlated with conflict control performance and neural processing of conflict resolution of stimulus–stimulus conflicts in the Flanker task. The fewer processing a task requires, the more likely there is an association between the T/C ratios and the behavioral and brain response, and the T/C ratios may not correlate with relatively underdeveloped cognitive control abilities. Furthermore, the dual-hormone effects on conflict resolution may be testosterone-driven in the Stroop and Flanker tasks, indicating the involvement of a testosterone-driven dual-hormone interaction during cortical maturation. These findings further supported the dual-hormone hypothesis and the dissociable processing mechanisms in varied conflict control tasks.

## Author Contributions

FS and TL designed the experiment, analyzed the data, and wrote the manuscript. XL and JS collected the data.

## Conflict of Interest Statement

The authors declare that the research was conducted in the absence of any commercial or financial relationships that could be construed as a potential conflict of interest.

## References

[B1] Abundis-Gutierrez A. Checa P. Castellanos C. Rueda M. R. (2014).Electrophysiological correlates of attention networks in childhood and early adulthood. *Neuropsychologia* 57 78–92. 10.1016/j.neuropsychologia.2014.02.013 24593898

[B2] Archer J. (2006). Testosterone and human aggression: an evaluation of the challenge hypothesis. *Neurosci. Biobehav. Rev.* 30 319–345. 10.1016/j.neubiorev.2004.12.007 16483890

[B3] Barrett-Connor E. Goodman-Gruen D. Patay B. (1999). Endogenous sex hormones and cognitive function in older men. *J. Clin. Endocrinol. Metab.* 84 3681–3685. 10.1210/jc.84.10.3681 10523014

[B4] Bexkens A. Van der Molen M. W. Annematt M. Huizenga H. M. (2014). Interference control in adolescents with Mild-to-Borderline Intellectual Disabilities and/or behavior disorders. *Child Neuropsychol.* 20 398–414. 10.1080/09297049.2013.799643 23755963

[B5] Bing O. Heilig M. Kakoulidis P. Sundblad C. Wiklund L. Eriksson E. (1998). High doses of testosterone increase anticonflict behaviour in rat. *Eur. Neuropsychopharmacol.* 8 321–323. 10.1016/S0924-977X(97)00095-3 9928924

[B6] Botvinick M. M. Cohen J. D. Carter C. S. (2004). Conflict monitoring and anterior cingulate cortex: an update. *Trends Cogn. Sci.* 8 539–546. 10.1016/j.tics.2004.10.003 15556023

[B7] Bryce D. Szûcs D. Soltész F. Whitebread D. (2011). The development of inhibitory control: an averaged and single-trial lateralized readiness potential study. *Neuroimage* 57 671–685. 10.1016/j.neuroimage.2010.12.006 21146618

[B8] Brydges C. R. Anderson M. Reid C. L. Fox A. M. (2013). Maturation of cognitive control: delineating response inhibition and interference suppression. *PLoS ONE* 8:e69826 10.1371/journal.pone.0069826 PMC3720932 23894548

[B9] Brydges C. R. Fox A. M. Reid C. L. Anderson M. (2014a). Predictive validity of the N2 and P3 ERP components to executive functioning in children: a latent-variable analysis. *Front. Hum. Neurosci.* 8:80 10.3389/fnhum.2014.00080 PMC3929846 24600376

[B10] Brydges C. R. Fox A. M. Reid C. L. Anderson M. (2014b). The differentiation of executive functions in middle and late childhood: a longitudinal latent-variable analysis. *Intelligence* 47 34–43. 10.1016/j.intell.2014.08.010

[B11] Crone E. A. Steinbeis N. (2017). Neural perspectives on cognitive control development during childhood and adolescence. *Trends Cogn. Sci.* 21 205–215. 10.1016/j.tics.2017.01.003 28159355

[B12] Crump M. J. Gong Z. Milliken B. (2006). The context-specific proportion congruent Stroop effect: location as a contextual cue. *Psychon. Bull. Rev.* 13 316–321. 10.3758/BF03193850 16893001

[B13] Davidson M. C. Amso D. Anderson L. C. Diamond A. (2006). Development of cognitive control and executive functions from 4 to 13 years: evidence from manipulations of memory, inhibition, and task switching. *Neuropsychologia* 44 2037–2078. 10.1016/j.neuropsychologia.2006.02.006 16580701 PMC1513793

[B14] Diamond A. Taylor C. (1996). Development of an aspect of executive control: development of the abilities to remember what I said and to “Do as I say, not as I do”. *Dev. Psychobiol.* 29 315–334. 10.1002/(SICI)1098-2302(199605)29:4<315::AID-DEV2>3.0.CO;2-T 8732806

[B15] Dismukes A. R. Johnson M. M. Vitacco M. J. Iturri F. Shirtcliff E. A. (2015). Coupling of the HPA and HPG axes in the context of early life adversity in incarcerated male adolescents. *Dev. Psychobiol.* 57 705–718. 10.1002/dev.21231 25213098 PMC5429593

[B16] Driscoll I. Resnick S. M. (2007). Testosterone and cognition in normal aging and Alzheimer’s disease: an update. *Curr. Alzheimer Res.* 4 33–45. 10.2174/156720507779939878 17316164

[B17] Eiland L. Romeo R. D. (2013). Stress and the developing adolescent brain. *Neuroscience* 249 162–171. 10.1016/j.neuroscience.2012.10.048 23123920 PMC3601560

[B18] Eriksen B. A. Eriksen C. W. (1974). Effects of noise letters upon identification of a target letter in a non-search task. *Percept. Psychophys.* 16 143–149. 10.3758/BF03203267

[B19] Fox N. A. Henderson H. A. Marshall P. J. Nichols K. E. Ghera M. M. (2005). Behavioral inhibition: linking biology and behavior within a developmental framework. *Annu. Rev. Psychol.* 56 235–262. 10.1146/annurev.psych.55.090902.141532 15709935

[B20] Frühholz S. Godde B. Finke M. Herrmann M. (2011). Spatio-temporal brain dynamics in a combined stimulus–stimulus and stimulus–response conflict task. *Neuroimage* 54 622–634. 10.1016/j.neuroimage.2010.07.071 20691791

[B21] Geschwind N. Galaburda A. M. (1985). Cerebral lateralization: biological mechanisms, associations, and pathology: I. A hypothesis and a program for research. *Arch. Neurol.* 42 428–459. 10.1001/archneur.1985.04060050026008 3994562

[B22] Glenn A. L. Raine A. Schug R. A. Gao Y. Granger D. A. (2011). Increased testosterone-to-cortisol ratio in psychopathy. *J. Abnorm. Psychol.* 120 389–399. 10.1037/a0021407 21133509 PMC3166523

[B23] Green H. J. Pakenham K. I. Headley B. C. Yaxley J. Nicol D. L. Mactaggart P. N. (2004). Quality of life compared during pharmacological treatments and clinical monitoring for non-localized prostate cancer: a randomized controlled trial. *BJU Int.* 93 975–979. 10.1111/j.1464-410X.2004.04763.x 15142146

[B24] Halari R. Simic M. Pariante C. M. Papadopoulos A. Cleare A. Brammer M. (2009). Reduced activation in lateral prefrontal cortex and anterior cingulate during attention and cognitive control functions in medication-naïve adolescents with depression compared to controls. *J. Child Psychol. Psychiatry* 50 307–316. 10.1111/j.1469-7610.2008.01972.x 19175815

[B25] Henry J. F. Sherwin B. B. (2012). Hormones and cognitive functioning during late pregnancy and postpartum: a longitudinal study. *Behav. Neurosci.* 126 73–85. 10.1037/a0025540 21928875 PMC4839972

[B26] Hermans E. J. Putman P. Baas J. M. Koppeschaar H. P. van Honk J. (2006). A single administration of testosterone reduces fear-potentiated startle in humans. *Biol. Psychiatry* 59 872–874. 10.1016/j.biopsych.2005.11.015 16458259

[B27] Hillman C. H. Buck S. M. Themanson J. R. Pontifex M. B. Castelli D. M. (2009). Aerobic fitness and cognitive development: event-related brain potential and task performance indices of executive control in preadolescent children. *Dev. Psychol.* 45 114–129. 10.1037/a0014437 19209995

[B28] Hommel B. (1993). The relationship between stimulus processing and response selection in the Simon task: evidence for a temporal overlap. *Psychol. Res.* 55 280–290. 10.1007/BF00419688

[B29] Janowsky J. S. (2006). Thinking with your gonads: testosterone and cognition. *Trends Cogn. Sci.* 10 77–82. 10.1016/j.tics.2005.12.010 16386941

[B30] Joëls M. Fernandez G. Roozendaal B. (2011). Stress and emotional memory: a matter of timing. *Trends Cogn. Sci.* 15 280–288. 10.1016/j.tics.2011.04.004 21571575

[B31] Johnstone S. J. Barry R. J. Markovska V. Dimoska A. Clarke A. R. (2009). Response inhibition and interference control in children with AD/HD: a visual ERP investigation. *Int. J. Psychophysiol.* 72 145–153. 10.1016/j.ijpsycho.2008.11.007 19095016

[B32] Jongen E. M. M. Jonkman L. M. (2008). The developmental pattern of stimulus and response interference in a color-object Stroop task: an ERP study. *BMC Neurosci.* 9:82 10.1186/1471-2202-9-82 PMC2535779 18775060

[B33] Jonkman L. M. (2006). The development of preparation, conflict monitoring and inhibition from early childhood to young adulthood: a Go/Nogo ERP study. *Brain Res.* 1097 181–193. 10.1016/j.brainres.2006.04.064 16729977

[B34] Kerns J. G. Cohen J. D. MacDonald A. W. Cho R. Y. Stenger V. A. Carter C. S. (2004). Anterior cingulated conflict monitoring and adjustments in control. *Science* 303 1023–1026. 10.1126/science.1089910 14963333

[B35] Khan N. A. Raine L. B. Drollette E. S. Scudder M. R. Kramer A. F. Hillman C. H. (2015). Dietary fiber is positively associated with cognitive control among prepubertal children. *J. Nutr.* 145 143–149. 10.3945/jn.114.198457 25527669 PMC4264019

[B36] Kornblum S. (1994). The way irrelevant dimensions are processed depends on what they overlap with: the case of Stroop-and Simon-like stimuli. *Psychol. Res.* 56 130–135. 10.1007/BF00419699 8008775

[B37] Kornblum S. Hasbroucq T. Osman A. (1990). Dimensional overlap – cognitive basis for stimulus-response compatibility – a model and taxonomy. *Psychol. Rev.* 97 253–270. 10.1037/0033-295X.97.2.253 2186425

[B38] Kornblum S. Stevens G. T. Whipple A. Requin J. (1999). The effects of irrelevant stimuli: the time course of stimulus-stimulus and stimulus-response consistency effects with Stroop-like stimuli, Simon-like tasks, and their factorial combinations. *J. Exp. Psychol. Hum. Percept. Perform.* 25 688–714. 10.1037/0096-1523.25.3.688

[B39] Larson M. J. Clayson P. E. Clawson A. (2014). Making sense of all the conflict: a theoretical review and critique of conflict-related ERPs. *Int. J. Psychophysiol.* 93 283–297. 10.1016/j.ijpsycho.2014.06.007 24950132

[B40] Liotti M. Woldorff M. G. Perez R. Mayberg H. S. (2000). An ERP study of the temporal course of the Stroop color-word interference effect. *Neuropsychologia* 38 701–711. 10.1016/S0028-3932(99)00106-2 10689046

[B41] Liu X. Park Y. Gu X. Fan J. (2010). Dimensional overlap accounts for independence and integration of stimulus-response compatibility effects. *Atten. Percept. Psychophys.* 72 1710–1720. 10.3758/APP.72.6.1710 20675812

[B42] Martin D. M. Burns N. R. Wittert G. (2009). Free testosterone levels, attentional control, and processing speed performance in aging men. *Neuropsychology* 23 158–167. 10.1037/a0014182 19254089

[B43] Mayr U. Awh E. Laurey P. (2003). Conflict adaptation effects in the absence of executive control. *Science* 6 450–452. 10.1038/nn1051 12704394

[B44] Mehta P. H. Josephs R. A. (2010). Testosterone and cortisol jointly regulate dominance: evidence for a dual-hormone hypothesis. *Horm. Behav.* 58 898–906. 10.1016/j.yhbeh.2010.08.020 20816841

[B45] Mehta P. H. Welker K. M. Zilioli S. Carré J. M. (2015). Testosterone and cortisol jointly modulate risk-taking. *Psychoneuroendocrinology* 56 88–99. 10.1016/j.psyneuen.2015.02.023 25813123

[B46] Moffat S. D. (2005). Effects of testosterone on cognitive and brain aging in elderly men. *Ann. N. Y. Acad. Sci.* 1055 80–92. 10.1196/annals.1323.014 16387720

[B47] Montoya E. Terburg D. Bos P. van Honk J. (2012). Testosterone, cortisol, and serotonin as key regulators of social aggression: a review and theoretical perspective. *Motiv. Emot.* 36 65–73. 10.1007/s11031-011-9264-3 22448079 PMC3294220

[B48] Mullane J. C. Corkum P. V. Klein R. M. McLaughlin E. (2009). Interference control in children with and without ADHD: a systematic review of Flanker and Simon task performance. *Child Neuropsychol.* 15 321–342. 10.1080/09297040802348028 18850349

[B49] Muller M. Aleman A. Grobbee D. E. de Haan E. H. van der Schouw Y. T. (2005). Endogenous sex hormone levels and cognitive function in aging men: is there an optimal level? *Neurology* 64 866–871. 10.1212/01.wnl.0000153072.54068.e3 15753424

[B50] Netherton C. Goodyer I. Tamplin A. Herbert J. (2004). Salivary cortisol and dehydroepiandrosterone in relation to puberty and gender. *Psychoneuroendocrinology* 29 125–140. 10.1016/S0306-4530(02)00150-6 14604596

[B51] Nguyen T. V. McCracken J. Ducharme S. Botteron K. N. Mahabir M. Johnson W. (2013). Testosterone-related cortical maturation across childhood and adolescence. *Cereb. Cortex* 23 1424–1432. 10.1093/cercor/bhs125 22617851 PMC3643718

[B52] Nigg J. T. (2000). On inhibition/disinhibition in developmental psychopathology: views from cognitive and personality psychology and a working inhibition taxonomy. *Psychol. Bull.* 126 220–246. 10.1037/0033-2909.126.2.220 10748641

[B53] Oei N. Y. L. Everaerd W. T. A. M. Elzinga B. M. Van Well S. Bermond B. (2006). Psychosocial stress impairs working memory at high loads: an association with cortisol levels and memory retrieval. *Stress* 9 133–141. 10.1080/10253890600965773 17035163

[B54] Perry P. J. Lund B. C. Arndt S. Holman T. Bever-Stille K. A. Paulsen J. (2001). Bioavailable testosterone as a correlate of cognition, psychological status, quality of life, and sexual function in aging males: implications for testosterone replacement therapy. *Ann. Clin. Psychiatry* 13 75–80. 10.1023/A:1016663523579 11534928

[B55] Perlstein W. M. Carter C. S. Barch D. M. Baird J. W. (1998). The Stroop task and attention deficits in schizophrenia: a critical evaluation of card and single-trial Stroop methodologies. *Neuropsychology* 12 414–425. 10.1037/0894-4105.12.3.414 9673997

[B56] Pfattheicher S. Keller J. (2014). Towards a biopsychological understanding of costly punishment: the role of basal cortisol. *PLoS ONE* 9:e85691 10.1371/journal.pone.0085691 PMC3885749 24416441

[B57] Pfeifer J. H. Allen N. B. (2012). Arrested development? Reconsidering dual-systems models of brain function in adolescence and disorders. *Trends Cogn. Sci.* 16 322–329. 10.1016/j.tics.2012.04.011 22613872 PMC3711850

[B58] Polich J. (2007). Updating P300: an integrative theory of P3a and P3b. *Clin. Neurophysiol.* 118 2128–2148. 10.1016/j.clinph.2007.04.019 17573239 PMC2715154

[B59] Ponzi D. Zilioli S. Mehta P. H. Maslov A. Watson N. V. (2016). Social network centrality and hormones: the interaction of testosterone and cortisol. *Psychoneuroendocrinology* 68 6–13. 10.1016/j.psyneuen.2016.02.014 26930262

[B60] Proctor R. W. Reeve T. G. (eds) (1990). *Stimulus-Response Compatibility: An Integrated Perspective*. Amsterdam: North-Holland.

[B61] Pruessner M. Hellhammer D. H. Pruessner J. C. Lupien S. J. (2003). Self-reported depressive symptoms and stress levels in healthy young men: associations with the cortisol response to awakening. *Psychosom. Med.* 65 92–99. 10.1097/01.PSY.0000040950.22044.10 12554820

[B62] Rueda M. R. Posner M. I. Rothbart M. K. Davis-Stober C. P. (2004). Development of the time course for processing conflict: an event-related potentials study with 4 year olds and adults. *BMC Neurosci.* 5:39 10.1186/1471-2202-5-39 PMC529252 15500693

[B63] Salminen E. K. Portin R. I. Koskinen A. Helenius H. Nurmi M. (2004). Associations between serum testosterone fall and cognitive function in prostate cancer patients. *Clin. Cancer Res.* 10 7575–7582. 10.1158/1078-0432.CCR-04-0750 15569988

[B64] Sapienza P. Zingales L. Maestripieri D. (2009). Gender differences in financial risk aversion and career choices are affected by testosterone. *Proc. Natl. Acad. Sci. U.S.A.* 106 15268–15273. 10.1073/pnas.0907352106 19706398 PMC2741240

[B65] Schoofs D. Preuss D. Wolf O. T. (2008). Psychosocial stress induces working memory impairments in an n-back paradigm. *Psychoneuendocrinology* 33 643–653. 10.1016/j.psyneuen.2008.02.004 18359168

[B66] Shangguan F. Shi J. (2009). Puberty timing and fluid intelligence: a study of correlations between testosterone and intelligence in 8- to 12-year-old Chinese boys. *Psychoneuroendocrinology* 34 983–988. 10.1016/j.psyneuen.2009.01.012 19249158

[B67] Sheridan M. Kharitonova M. Martin R. E. Chatterjee A. Gabrieli J. D. (2014). Neural substrates of the development of cognitive control in children ages 5-10 years. *J. Cogn. Neurosci.* 26 1840–1850. 10.1162/jocn_a_00597 24650280 PMC4080218

[B68] Sherman G. D. Lerner J. S. Josephs R. A. Renshon J. Gross J. J. (2016). The interaction of testosterone and cortisol is associated with attained status in male executives. *J. Pers. Soc. Psychol.* 110 921–929. 10.1037/pspp0000063 26302434

[B69] Shields G. S. Bonner J. C. Moons W. G. (2015). Does cortisol influence core executive functions? A meta-analysis of acute cortisol administration effects on working memory, inhibition, and set-shifting. *Psychoneuroendocrinology* 58 91–103. 10.1016/j.psyneuen.2015.04.017 25973565

[B70] Simon J. R. (1969). Reactions toward the source of stimulation. *J. Exp. Psychol.* 81 174–176. 10.1037/h0027448 5812172

[B71] Simon J. R. Small A. M. Jr. (1969). Processing auditory information: interference from an irrelevant cue. *J. Appl. Psychol.* 53 433–435. 10.1037/h0028034 5366316

[B72] SizoNenko P. C. Paunier L. (1975). Hormonal changes in puberty III: Correlation of plasma dehydroepiandrosterone, testosterone, FSH, and LH with stages of puberty and bone age in normal boys and girls and in patients with Addison’s disease or hypogonadism or with premature or late adrenarche. *J. Clin. Endocrinol. Metab.* 41 894–904. 10.1210/jcem-41-5-894 127002

[B73] Smith J. L. Johnstone S. J. Barry R. J. (2008). Movement-related potentials in the Go/Nogo task: the P3 reflects both cognitive and motor inhibition. *Clin. Neurophysiol.* 119 704–714. 10.1016/j.clinph.2007.11.042 18164657

[B74] Spillers G. J. Unsworth N. (2011). Variation in working memory capacity and temporal-contextual retrieval from episodic memory. *J. Exp. Psychol. Learn. Mem. Cogn.* 37 1532–1539. 10.1037/a0024852 21823812

[B75] Stanton S. J. Liening S. H. Schultheiss O. C. (2011). Testosterone is positively associated with risk taking in the Iowa Gambling Task. *Horm. Behav.* 59 252–256. 10.1016/j.yhbeh.2010.12.003 21167166

[B76] Stroop J. R. (1935). Studies of interference in serial verbal reactions. *J. Exp. Psychol.* 18 643–662. 10.1037/h0054651

[B77] Terburg D. Morgan B. Van Honk J. (2009). The testosterone-cortisol ratio: a hormonal marker for proneness to social aggression. *Int. J. Law Psychiatry* 32 216–223. 10.1016/j.ijlp.2009.04.008 19446881

[B78] Tillman C. M. Wiens S. (2011). Behavioral and ERP indices of response conflict in Stroop and flanker tasks. *Psychophysiology* 48 1405–1411. 10.1111/j.1469-8986.2011.01203.x 21457276

[B79] Tops M. Boksem M. A. S. (2011). Cortisol involvement in mechanisms of behavioral inhibition. *Psychophysiology* 48 723–732. 10.1111/j.1469-8986.2010.01131.x 20874754

[B80] Tops M. Boksem M. A. S. Wester A. E. Lorist M. M. Meijman T. F. (2006). Task engagement and the relationships between the error-related negativity, agreeableness, behavioral shame proneness and cortisol. *Psychoneuroendocrinology* 31 847–858. 10.1016/j.psyneuen.2006.04.001 16774808

[B81] Treccani B. Cubelli R. Della Sala S. Umilta C. (2009). Flanker and Simon effects interact at the response selection stage. *Q. J. Exp. Psychol.* 62 1784–1804. 10.1080/17470210802557751 19180364

[B82] Tsujimoto S. (2008). The prefrontal cortex: functional neural development during early childhood. *Neuroscientist* 14 345–358. 10.1177/1073858408316002 18467667

[B83] Unsworth N. Brewer G. A. Spillers G. J. (2011). Variation in working memory capacity and forgetting over both the short and the long term: an application of the population dilution model. *J. Cogn. Psychol.* 23 243–255. 10.1080/20445911.2011.493153

[B84] van der Molen M. W. (2000). Developmental changes in inhibitory processing: evidence from psychophysiological measures. *Biol. Psychol.* 54 207–239. 10.1016/S0301-0511(00)00057-0 11035224

[B85] van Honk J. Harmon-Jones E. Morgan B. E. Schutter D. J. L. G. (2010). Socially explosive minds: the triple imbalance hypothesis of reactive aggression. *J. Pers.* 78 67–94. 10.1111/j.1467-6494.2009.00609.x 20433613

[B86] van Honk J. Schutter D. J. L. G. (2006). Unmasking feigned sanity: a neurobiological model of emotion processing in primary psychopathy. *Cogn. Neuropsychiatry* 11 285–306. 10.1080/13546800500233728 17354073

[B87] van Honk J. Schutter D. J. L. G. Hermans E. J. Putman P. (2003). Low cortisol levels and the balance between punishment sensitivity and reward dependency. *Neuroreport* 14 1993–1996. 10.1097/00001756-200310270-00023 14561936

[B88] van Meel C. S. Heslenfeld D. J. Rommelse N. N. Oosterlaan J. Sergeant J. A. (2012). Developmental trajectories of neural mechanisms supporting conflict and error processing in middle childhood. *Dev. Neuropsychol.* 37 358–378. 10.1080/87565641.2011.653062 22612547

[B89] van Strien J. W. Weber R. F. A. Burdorf A. Bangma C. (2009). Higher free testosterone level is associated with faster visual processing and more flanker interference in older men. *Psychoneuroendocrinology* 34 546–554. 10.1016/j.psyneuen.2008.10.020 19042092

[B90] van Veen V. Carter C. S. (2002). The anterior cingulate as a conflict monitor: fMRI and ERP studies. *Physiol. Behav.* 77 477–482. 10.1016/S0031-9384(02)00930-7 12526986

[B91] Ventura T. Gomes M. C. Carreira T. (2012). Cortisol and anxiety response to a relaxing intervention on pregnant women awaiting amniocentesis. *Psychoneuroendocrinology* 37 148–156. 10.1016/j.psyneuen.2011.05.016 21705148

[B92] Wang Z. Zhou R. Shah P. (2014). Spaced cognitive training promotes training transfer. *Front. Hum. Neurosci.* 8:217 10.3389/fnhum.2014.00217 PMC3989588 24782744

